# A Review of Current Approaches to Pain Management in Knee Osteoarthritis with a Focus on Italian Clinical Landscape

**DOI:** 10.3390/jcm13175176

**Published:** 2024-08-31

**Authors:** Stefano Giaretta, Alberto Magni, Alberto Migliore, Silvia Natoli, Filomena Puntillo, Gianpaolo Ronconi, Luigi Santoiemma, Cristiano Sconza, Ombretta Viapiana, Gustavo Zanoli

**Affiliations:** 1 UOC Ortopedia e Traumatologia OC San Bortolo di Vicenza (AULSS 8 Berica), 36100 Vicenza, Italy; 2 Local Health Department, Desenzano sul Garda, 25015 Brescia, Italy; alberto.magni@icloud.com; 3 Unit of Rheumatology, San Pietro Fatebenefratelli Hospital, 00189 Rome, Italy; migliore.alberto60@gmail.com; 4 Department of Clinical-Surgical, Diagnostic, and Pediatric Sciences, University of Pavia, 27100 Pavia, Italy; silvia.natoli@unipv.it; 5 Pain Unit, IRCCS Policlinico San Matteo, 27100 Pavia, Italy; 6 Anaesthesia, Intensive Care and Pain Unit, Department of Interdisciplinary Medicine, University of Bari “Aldo Moro”, 70124 Bari, Italy; filomena.puntillo@uniba.it; 7 Department of Rehabilitation, Catholic University of the Sacred Heart, 00168 Rome, Italy; gianpaolo.ronconi@policlinicogemelli.it; 8 Local Health Department Bari, Modugno, 70026 Bari, Italy; santoiemmaluigi@libero.it; 9 IRCCS Humanitas Research Hospital, Rozzano, 20089 Milan, Italy; cristiano.sconza@humanitas.it; 10 Rheumatology Unit, Azienda Ospedaliera Universitaria Integrata di Verona, University of Verona, 37126 Verona, Italy; ombretta.viapiana@univr.it; 11 Orthopaedic Ward, Casa di Cura Santa Maria Maddalena, Occhiobello, 45030 Rovigo, Italy; zanolig@me.com

**Keywords:** osteoarthritis, knee osteoarthritis, intra-articular therapy, NSAIDs, total knee arthroplasty, treat-to-target treatment, individualized medicine

## Abstract

The global cases of knee osteoarthritis (KOA) are projected to increase by 74.9% by 2050. Currently, over half of patients remain dissatisfied with their pain relief. This review addresses unmet needs for moderate-to-severe KOA pain; it offers evidence and insights for improved management. Italian experts from the fields of rheumatology, physical medicine and rehabilitation, orthopedics, primary care, and pain therapy have identified several key issues. They emphasized the need for standardized care protocols to address inconsistencies in patient management across different specialties. Early diagnosis is crucial, as cartilage responds better to early protective and structural therapies. Faster access to physiatrist evaluation and reimbursement for physical, rehabilitative, and pharmacological treatments, including intra-articular (IA) therapy, could reduce access disparities. Concerns surround the adverse effects of oral pharmacological treatments, highlighting the need for safer alternatives. Patient satisfaction with corticosteroids and hyaluronic acid-based IA therapies reduces over time and there is no consensus on the optimal IA therapy protocol. Surgery should be reserved for severe symptoms and radiographic KOA evidence, as chronic pain post-surgery poses significant societal and economic burdens. The experts advocate for a multidisciplinary approach, promoting interaction and collaboration between specialists and general practitioners, to enhance KOA care and treatment consistency in Italy.

## 1. Introduction

Osteoarthritis (OA) is a common and debilitating condition characterized by dynamic structural alterations that affect the entire joint, from cartilage to bone and synovial membranes, leading to the loss of normal joint function [1].

Global OA prevalence is rising substantially, with a 132.2% increase in cases from 1990 to 2020, with 595 million cases reported in 2020 [2]. Prevalence increases with age [2], except in knee OA (KOA), which peaks at the age of 80–84 years and then decreases [2]. The knee is the most commonly affected site [3], with a prevalence of around 365 million cases in 2019 [3], with more cases occurring in women [2,3]. By 2050, global cases of KOA are projected to increase by 74.9%, hand OA by 48.6%, hip OA by 78.6%, and other types of OA by 95.1% [2].

In Italy, the prevalence of OA was reported as 18.8% in 2021, showing a rising trend since 2012, when it was at 16.8% [4]. The distribution of cases among sexes and age aligns with the global data [4].

Risk factors for KOA include female gender [5], obesity [6], previous knee injury [7], knee malalignment [8], and knee extensor muscle weakness [9]. Despite its prevalence in older adults [5], there is a significant burden among those under 65 years [10,11], particularly individuals with knee injuries [12]. Pain and stiffness in the affected joint are the leading symptoms of KOA [13]. During the initial stages of KOA, pain is typically provoked by load-bearing, but as the condition progresses, it tends to become more persistent and constant [14]. The progression of pain and the level of motor disability in individuals with KOA vary greatly, based on the severity and scope of degenerative changes in the knee [15,16], and may correlate with either the linear progression of the disease or the radiographic advancement of OA [17]. However, the radiographic severity of KOA degeneration is not a reliable predictor of pain intensity and severity [18].

Disability related to KOA is highly influenced by the presence and severity of pain, which detrimentally affects mobility and overall quality of life (QoL), limiting social engagement and sleep quality [19]. The prevalence of symptomatic KOA varies from 7% to 16% in studies [20,21,22,23], although data from a multinational Adelphi OA Disease Specific Programme (1547 participants) showed that 54% of European participants with hip OA or KOA reported living with moderate or severe pain [24]. Epidemiological studies are needed in Italy to better understand the pain associated with KOA. KOA pain is primarily nociceptive in origin, arising from the mechanical activation of nociceptors due to abnormal loading of a damaged joint, as well as from nociceptors that are activated and sensitized by inflammation [25]. Additionally, some authors argue that nerve damage may occur in the injured joint, suggesting the involvement of a neuropathic pain mechanism [25]. The literature suggests that between 20% and 67% of patients with KOA experience neuropathic pain [26,27,28,29].

Accurate differential diagnosis and the recognition of these various factors are essential for determining the most suitable therapeutic approach [30].

OA has also significant economic implications, resulting in considerable health resources utilization, and substantial direct and indirect costs [31], including loss of productivity [32].

With these insights in mind, this comprehensive review aims to explore the unmet needs of individuals experiencing moderate to severe KOA pain, informing both current and future strategies for KOA management and treatment.

## 2. Methods

The authors, who are 10 Italian experts from diverse fields (i.e., rheumatology, physical medicine and rehabilitation, orthopedics, primary care, and pain therapy), conducted a comprehensive non-systematic review of the existing literature on PubMed/MEDLINE and Google Scholar without the restriction of time, using the following keywords: “knee osteoarthritis” AND “pain management” OR “costs” OR “rehabilitation” OR “exercise” OR “weight loss” OR “conservative treatment” OR “intra-articular injections” OR “prolotherapy” OR “platelet-rich plasma” OR “mesenchymal cells” OR “chondrocyte implantation” OR “paracetamol” OR “SYSADOAs” OR “NSAIDs” OR “opioids” OR “duloxetine” OR “surgical treatment” OR “UKA” OR “TKA” OR “treat-to-target” OR “innovative treatments” OR “TRPV-1”. All types of study designs were included to expand the scope of this narrative review. References of the articles were screened by title and abstracts to identify relevant information on these topics. Further full-text screening was performed for previously published review articles to identify gaps in the selected literature and elaborate on the importance of this topic comprehensively.

Then the authors discussed the state of the art in KOA management.

## 3. Clinical and Economic Impact of Osteoarthritis-Associated Pain

The lifetime risk of symptomatic KOA ranges from 14% to 45% [10,23], rising to 60.5% for obese individuals [23]. Severe OA symptoms are linked to reduced health-related QoL and difficulties in mobility and self-care [33]. Symptomatic individuals experience faster gait decline over time [34].

Due to a lack of physical activity, OA patients are more prone to develop cardiovascular disease [35], hypercholesterolemia [36], hypertension [37], diabetes [38], and related overall mortality [39].

OA patients, specifically those with KOA, often seek medical attention for characteristic pain [18] that evolves from intermittent, activity-related discomfort to chronic pain characterized by peripheral and central pain sensitization [25]. This pain varies in intensity [40] and qualities [41], such as aching, dull, sharp, and stubbing, as well as burning, tingling, numbness, or pins and needles, and is highly disabling. The chronic pain experience bears a high personal and social impact, being a “difficult to treat” condition that deserves a multi-professional approach, including psychosocial interventions [42].

Identifying and validating distinct molecular and clinical OA, and particularly KOA, phenotypes, such as biomechanical, post-menopausal estrogen deficiency-related, metabolic, inflammatory, chronic pain, metabolic syndrome, bone, cartilage metabolism, mechanical overload, and minimal joint disease phenotypes [43,44,45], is crucial for guiding optimal therapy [44]. Non-pharmacological therapies benefit biomechanical OA [43], while inflammatory and symptomatic OA may respond to anti-inflammatory drugs [43]. Weight loss is effective in overweight patients [46,47], who may also benefit from anti-lipidemic drugs or caloric restriction [43]. Diabetes may contribute to the high prevalence of OA, being an independent risk factor for joint degeneration and faster worsening of pain [48].

Effective and long-lasting treatments are a high priority to reduce the social and personal costs of KOA. Indeed, whereas healthcare costs are negatively impacted by knee joint replacements, indirect social (work loss, premature retirement) and healthcare costs (reduced mobility, chronic pain) in persons refusing or being excluded from surgery due to comorbidities, and personal costs (reduced productivity, low income) also contribute to the overall disease burden [49]. The economic burden of OA exceeded EUR 1000 per year in incremental healthcare costs in 2011 [31], increasing with age and disease severity and peaking with surgery [31,50]. The demand for primary knee replacements in the USA is expected to surge by 673%, reaching 3.48 million procedures by 2030 [50], while an increase of 45% is expected for 2050 in Italy [51], a trend comparable to that expected in other European countries [52,53,54]. OA-related costs can represent 0.25% to 0.5% of a country’s gross domestic product [31].

## 4. Challenges in Choosing the Right Approach for Treating Osteoarthritis

The objective in treating KOA is to alleviate pain, enhance function, and improve QoL through non-pharmacological, pharmacological, and surgical interventions, depending on the stage of the disease, aligning with guideline recommendations [55,56,57,58,59]. However, therapy selection is challenging due to the absence of standardized approaches and the heterogeneity among available guidelines [55,56,57,58,60,61,62]. These guidelines [55,56,57,58,60,61,62] lack clarity, especially regarding the management of persistent chronic symptoms. Table 1 highlights the discrepancies among guidelines in the levels of recommendation for oral SYSADOAs (Table 1), while Table 2 does the same for intra-articular (IA) hyaluronic acid (HA) and IA corticosteroids (Table 2). Furthermore, guidelines [55,56,57,58,60,61,62] do not provide homogeneous recommendations on how to manage flares and inadequately address the common practice of a multimodal yet patient-tailored approach, as highlighted in a study by Veronese et al. [63]. This results in variations in treatment and patients’ confusion over which specialist to consult for managing KOA, contributing to inconsistent care paths and non-adherence [63,64,65] and prompting the need for adequate education of healthcare professionals in managing KOA [66].

Personalized care with a multidisciplinary approach accounting for age, comorbidities, concurrent treatments, and pain levels is essential [64]. Advanced omics technologies can identify early molecular changes preceding structural alterations [68,69], such as chondrosenescence, mitochondrial alterations, secretome, micro-environmental changes, and inflammation [44].

MRI studies often detect cartilage, synovial membrane, and subchondral bone involvement before radiographic evidence emerges [70], depicting a stage of disease that may be responsive to preventative non-pharmacological treatments [64]. Synovial inflammation contributes to OA symptoms [71], and imaging, histopathological findings, and MRI aid in identifying patients with aggressive synovitis and late-stage OA changes in the knee [72]. Integrating imaging and biochemical markers to identify molecular endotypes and correlate them with clinical phenotypes can aid in predictive modeling for individualized treatments [44].

Many drugs cannot be used for a long time due to their adverse effects and incompatibility with medications used for KOA-associated comorbidities, highlighting the need for newer therapeutic options [73,74,75,76]. Other treatments, such as HA injections and genicular nerve ablation, have produced conflicting results, are weakly recommended or not considered in some major guidelines [56,58,60,61] (Table 2 and Table 3), and they are not reimbursed in many geographies [77]. Regrettably, patients’ expectations are often overlooked despite growing research highlighting the significance of a patient-centered approach [78,79].

### 4.1. Conservative Management of Knee Osteoarthritis

#### 4.1.1. Non-Pharmacological Approaches for Knee Osteoarthritis Management

The European Society for Clinical and Economic Aspects of Osteoporosis, Osteoarthritis, and Musculoskeletal Diseases (ESCEO) strongly endorses non-pharmacological strategies, including a healthy diet, weight management, physical activity, and patient education across different KOA severities [55,57]. Evidence shows that proper exercise therapy may postpone total joint replacement [59].

A multi-pronged OA treatment approach involves healthcare professionals such as rheumatologists, physiatrists, endocrinologists, psychologists, orthopedic technicians, and dietitians [80]. Due to the frailty and comorbidities [81] common in OA patients, collaboration with specialists, such as neurologists, geriatrists, pain therapists and internists is frequently necessary [64].

General practitioners (GPs) play a vital role in the initial management of ordering diagnostic imaging [65]. In more severe cases, a surgical consultation will be necessary [65]. When non-surgical options are suitable, the physiatrist could play a central role within the rehabilitation team, taking a holistic, person-centered approach and collaborating with GPs and pain therapists to create individualized rehabilitation plans [55,59,64,80,82] addressing modifiable risk factors and therapeutic goals [64,65]. In other settings, this role can be played by a rheumatologist, an orthopedic surgeon, or a physiotherapist.

Rehabilitation is suggested as the first-line treatment for OA, including KOA, in many guidelines [57,58,61], and it is considered safer than pharmacological interventions [60,83].

In addition to weight reduction, various interventions can modify knee overload. These include plantar orthoses, knee braces, walking aids, and education on joint care. A recent randomized crossover trial found that a knee brace with valgus and external rotation functions was more effective than foot orthoses in reducing pain and knee adduction moment after 3 months [84]. Unloader braces are effective by decreasing the heightened activity and co-contraction of the periarticular knee muscles in medial KOA [85]. Patients with KOA exhibit altered pressure patterns while walking [86], and lateral-wedge insoles might prevent the deterioration of plantar pressure in these patients [87]. However, most evidence does not support the effectiveness of lateral wedged or other insoles in reducing pain or improving function in KOA [59]. Nonetheless, updated EULAR recommendations advise the use of comfortable shoes with ample space for the toes during weight-bearing for individuals with hip and knee OA [59]. Walking aids such as using a crutch or cane in the opposite hand or wheeled walkers for bilateral KOA, especially in elderly patients, may also help alleviate pain [88], and their use is recommended by some guidelines [58,61,62]. Additionally, a recent systematic review reported that education programs significantly improve pain and function, even as standalone interventions, and positively enhance the outcomes of conservative treatments [89].

Strength training exercises are crucial for addressing KOA, enhancing muscle power and joint stability, reducing pain, and improving overall QoL [90]. Combining these exercises with polyvagal exercises shows synergistic effects, yielding superior outcomes in managing symptoms, reducing pain, and fostering relaxation [91]. In patients with KL grade 2 KOA, this integrated approach surpasses relying solely on strength training, resulting in greater reductions in joint pain, stiffness, and functional limitations, along with notable improvements in psychological and social domains [91]. Since the COVID-19 pandemic, telehealth-based exercise interventions have been widely used, demonstrating effectiveness in alleviating KOA pain [92]. When delivered as a smartphone app, they have also shown improvement in physical function [92].

Physical Therapeutic Means including therapeutic ultrasound, low-intensity pulsed ultrasound, low-intensity laser, high-intensity laser, and even shock waves, can have a favorable effect on the multimodal treatment of KOA, in the initial stages of Rehabilitation. Therapeutic ultrasound has been an effective and safe treatment option for alleviating pain and enhancing function in individuals with KOA [93], especially when used in conjunction with other physical therapy regimens [94]. One particular study demonstrated that continuous long-duration low-intensity ultrasound significantly decreased pain and improved joint function in patients experiencing moderate to severe KOA pain [95]. Low-intensity pulsed ultrasound (LIPUS) is also a promising non-pharmacological therapy within KOA rehabilitation programs, as it can substantially reduce pain and aid functional recovery [96]. Notably, LIPUS can be more effective than continuous ultrasound [97,98,99], although its widespread use is limited by high costs the need for specialized training, and the intensive time-schedule [100]. Current guidelines do not yet include recommendations for LIPUS in treating KOA.

Low-level laser therapy has been found to decrease the need for pain medication and improve performance in the sit-to-stand test [101], as well as enhance muscle strength and function in KOA patients [102]. Various randomized clinical trials have supported LLLT as a reliable non-pharmacological and non-surgical treatment for KOA, often in combination with exercise, suggesting its potential as a viable therapy [103]. However standardized protocols based on clinical evidence are required for its broader clinical application [103]. Recent studies have highlighted the increased effectiveness of high-intensity laser therapy (HILT) in reducing pain and disability in KOA patients compared to conventional physiotherapies and LLLT [104,105]. Extracorporeal shockwave therapy has also emerged as a safe and effective treatment for mild KOA, offering pain relief and improved function [106,107,108,109]. Additionally, it has been suggested that it can lead to objective improvements observable through ultrasonography [110].

A promising non-pharmacological approach for chronic pain relief in KOA involves percutaneous neuromodulation, utilizing low- or medium-frequency current through a puncture needle to stimulate peripheral nerves or muscles [111]. This method demonstrates pain reduction, improved function, and decreased analgesic use in KOA patients [112,113,114] with minimal side effects [111]. The ultimate goal is achieving the Patient Acceptable Symptom State (PASS) and effectively managing symptoms, enabling patients to perform daily activities satisfactorily [115,116]. This necessitates personalized therapy, requiring vigilant and timely adjustments for each patient, presenting a challenge for healthcare providers [115,116] due to each patient’s uniqueness [79].

Table 3 summarizes the pros and cons of non-pharmacological KOA, along with opinions from some major scientific societies.

**Table 3:** Non-pharmacological treatments in KOA patients without comorbidities: pros, cons, and guideline recommendations. Source: original.

Non-Pharmacological Treatments	Level of Recommendation *
Joint specific education	
Pros Improve pain and function Enhance the outcomes of conservative treatments	OARSI [56]: Strong (Core) ESCEO [57]: Strong ACR/AF [58]: Strong EULAR [59]: A NICE [61]: Recommended SIOT [62]: Strong
Weight management	
Pros Reduces risks for symptomatic KOA and pain Improves functional, clinical, and surgical outcomes	OARSI [56]: Strong ESCEO [55]: Strong ACR/AF [58]: Strong EULAR [60]: B NICE [61]: Recommended SIOT [62]: Strong
Exercise therapy	
Pros Potential to delay the need for total joint replacement. Enhances muscle power, function and pain reduction through strength training exercise. Synergistic effects when combined with strengthening exercises (e.g., polyvagal exercises) Safer than pharmacological interventions (e.g., rehabilitation). Pain reduction through tele-health exercise programs Cons High costs	OARSI [56]: Strong ESCEO [55]: Strong ACR/AF [58]: Strong EULAR [60]: A NICE [61]: Recommended SIOT [62]: Strong
Walking aids	
Pros May help reduce pain May lower the risk of falls	OARSI [56]: No recommendation ESCEO [55]: No recommendation ACR/AF [58]: Strong (cane) EULAR [59]: A NICE [61]: Cane recommended; other devices (e.g., insoles, braces) recommended only for abnormal loading or joint instability after exercise failure SIOT [62]: Strong (for symptomatic KOA)
Therapeutic ultrasound (continuous and pulsed)	
Pros Potential benefits for pain and function, especially when combined with other physical therapies Cons High costs (e.g., LIPUS) Requires specialized training (e.g., LIPUS) Intensive time commitment	OARSI [56]: No recommendation ESCEO [55]: No recommendation ACR/AF [58]: Conditional EULAR [60]: C NICE [61]: Not recommended SIOT [62]: No recommendation
Laser therapy (LLLT and HILT)	
Pros Reduces the need for pain medication Improves function Potentially more effective than conventional physical therapies Cons Lack of standardized protocols	OARSI [56]: No recommendation ESCEO [55]: No recommendation ACR/AF [58]: No recommendation EULAR [60]: B NICE [61]: Not recommended SIOT [62]: No recommendation
Extracorporeal shockwave therapy	
Pros Improves pain and function	OARSI [56]: No recommendation ESCEO [55]: No recommendation ACR/AF [58]: No recommendation EULAR [60]: No recommendation NICE [61]: No recommendation SIOT [62]: No recommendation
Peripheral nerve and muscle modulation	
Pros Improves pain and function Reduces analgesics use Cons Mild short-term side effects	OARSI [56]: No recommendation ESCEO [55]: No recommendation ACR/AF [58]: Strong against EULAR [60]: No recommendation NICE [61]: NOT recommended SIOT [62]: NOT recommended

HA: hyaluronic acid; OARSI: Osteoarthritis Research Society International; ESCEO: European Society for Clinical and Economic Aspects of Osteoporosis, Osteoarthritis, and Musculoskeletal Diseases; ACR: American College of Rheumatology; AF: Arthritis Foundation; EULAR: European League Against Rheumatism; NICE: National Institute for Health and Care Excellence; SIOT: Italian Orthopaedic and Traumatology Society. * In the absence of comorbidities.

#### 4.1.2. Topical and Oral Pharmacological Approaches for Knee Osteoarthritis Management

Paracetamol is a therapeutic option for KOA management but is less effective in reducing inflammatory pain compared to NSAIDs [117], having a weak inhibitory effect on cyclooxygenase-1 (COX-1) and COX-2 [64]. Furthermore, within inflamed tissues, free radicals deactivate paracetamol, rendering it ineffective in targeting COX-2 [118]. Paracetamol is recommended for short-term rescue analgesia when NSAIDs are contraindicated [55,56,58]. However, concerns about its safety profile have emerged due to various gastrointestinal, cardiovascular, hepatic, and renal toxicity adverse events [119].

Oral symptomatic slow-acting drugs for OA (SYSADOAs), including glucosamine hydrochloride (GlcNH), glucosamine sulfate (GlcNS), chondroitin sulfate (CS), diacerein, unsaponifiable soy extract, and avocado extract, lack analgesic properties [120]. Their effects emerge after 2–3 weeks of prolonged administration, providing symptomatic pain relief lasting for months [120], unlike NSAIDs, which require continuous administration [121]. CS and glucosamine (GlcN) demonstrate high efficacy in improving OA parameters [122,123,124], with a hypothesized mechanism involving NF-kB inhibition [120]. Despite a good safety profile [123], the varying efficacy of different formulations hinders a unanimous consensus among international societies. Both ESCEO and an Italian committee recommend pharmaceutical-grade prescription glucosamine (pCGS) and CS as a first-line option [55,125]. Boswellia, curcumin extracts, avocado unsaponifiable extract, and diacerein lack therapeutic endorsement due to insufficient clinical evidence [120], prompting ESCEO to advocate SYSADOA formulations with proven efficacy and safety data for OA treatment in current clinical practice [126]. Other scientific societies provide contrasting recommendations [58,60,62], highlighting the need for more convincing evidence. NSAIDs inhibit prostaglandins, providing peripheral sensitization relief [64]. NSAID therapy choice for KOA is influenced by factors, such as patient age, comorbidities, polypharmacy, and drug considerations, including efficacy, safety regarding cardiovascular events, gastrointestinal bleeding, nephrotoxicity, potential interactions, and formulation [64]. Thus, low-dose NSAIDs are recommended for a restricted period as a first-line treatment for managing inflammatory episodes [55,56,58,80].

Topical NSAIDs, available in the form of creams, gels, sprays, or patches applied to the skin, are advisable for individuals with symptomatic KOA and concurrent medical conditions [62]. The choice between topical or oral NSAIDs is influenced by the patient’s risk profile [57]. Oral NSAIDs are recommended for patients with persistent symptoms despite appropriate background therapy [55,57] with intermittent or extended cycles based on the patient’s risk profile [55]. In particular, the use of oral NSAIDs, ideally coupled with a proton pump inhibitor or selective COX-2 inhibitors [57], is recommended for individuals without comorbidities or with gastrointestinal ailments [57,60]. While they are comparable to SYSADOAs, they may be more suitable for severe pain or when SYSADOAs are ineffective [55].

Opioids are mentioned as effective in reducing pain in several guidelines [55,56,58,61]; however, they are burdened by several adverse effects in the long term, such as constipation, low bone density due to sexual hormone-axis suppression, tolerance, and misuse [127,128]. Short-term side effects include drowsiness, dizziness, nausea, constipation, and an increase in the risk of falls [55,58,60,61,62,119]. The Osteoarthritis Research Society International OARSI advises against the use of opioids for KOA patients who have long disease duration and are at risk of developing tolerance [56,57], while other societies suggest weak opioids as a temporary measure while patients await surgical treatment, particularly if NSAIDs prove ineffective against pain [55,58,60,61,62,119].

For patients with central sensitization, widespread pain, and/or depression, treatment with duloxetine, a serotonin–norepinephrine reuptake inhibitor, is recommended by international guidelines as an alternative to opioids [57,58,60,62].

Table 1 summarizes the pros and cons of topical and oral pharmacological treatments for KOA, along with opinions from some major scientific societies.

### 4.2. Minimally Invasive Pharmacological and Non-Pharmacological Approaches for the Treatment of KOA

IA treatments, recommended by ESCEO, OARSI, and Italian guidelines, are common for established KOA [55,56,57,58,129]. This approach offers specific advantages, including enhanced local bioavailability, minimized systemic exposure, and reduced frequency of adverse effects, which are minimally transient and primarily local [130]. However, effectiveness is typically limited to the early or middle stages of the disease [131]; administration demands specific skills and experience and is contraindicated in case of skin or systemic infections, hemorrhagic disorders, and uncontrolled diabetes [130].

Currently, the primary focus of IA therapy revolves around corticosteroids and HA preparations, both approved by the US Food and Drug Administration (FDA) and the European Medicines Agency (EMA) [130]. Nonetheless, other alternative IA therapies deserve to be mentioned, including those involving ozone and botulinum toxin, mesenchymal cells (MSCs), platelet-rich plasma (PRP), and dextrose. All these therapeutic options exhibit anti-inflammatory and/or chondroprotective and regenerative potential [132,133,134,135,136].

Corticosteroids, such as triamcinolone provide rapid pain relief and efficacy in cases of synovitis [137,138]. However, the therapeutic effect of corticosteroids typically lasts only up to approximately 2–4 weeks [62,130] with associated risks in repeated and long-term use (>6 weeks) [139,140]. Societies recommend restricting corticosteroid use to the short term; the Italian Orthopaedic and Traumatology Society (SIOT) recommends corticosteroid use for treating acute exacerbations once a week, with a maximum duration of 3 weeks [62].

IA steroid injections can be followed by viscosupplementation with IA HA [141], which has a more favorable long-term safety profile [142] and is suitable for individuals with insufficient pain relief from oral medications (such as NSAIDs and paracetamol), exercise, and physical therapy or who have pre-existing renal or gastrointestinal intolerance to NSAIDs [143]. HA has demonstrated chondroprotective, analgesic, and anti-inflammatory actions [144]. SIOT recommends administering IA HA once a week for 2–4 weeks [62]. This treatment can be repeated after 12 months for patients without knee swelling or flares [62]. Various HA preparations with different molecular weights are currently employed in IA treatment [137]. Research indicates that HA preparations exhibit a positive effect between 4 and 24 weeks post-injection [138]. However, due to the inconsistent guideline recommendations that suggest IA HA as a stage 2 or 3 option after the failure of core treatments or in cases with NSAID contraindications [57,58], many international public healthcare systems do not reimburse IA HA treatment.

Intra-articular injections of ozone and botulinum toxin have recently been studied for their interesting anti-inflammatory and analgesic therapeutic properties while maintaining a high safety profile, although there is not yet sufficient scientific evidence for their standardized use [145,146].

Adipose, bone marrow, or umbilical cord-derived MSCs exhibit therapeutic potential for pain reduction and joint improvement [147]. However, further clinical trials with larger samples and longer follow-ups are necessary to confirm the efficacy of MSC transplantation for osteoarthritis treatment.

PRP injections are a treatment option for pain reduction in KOA and improvement of function up to 12 months of follow-up [148], even if the beneficial effect does not correlate with radiographic or MRI improvement [149]. PRP injections were associated with a median delay of surgery of 4 years in over 1000 patients with KOA [150]. Studies with longer follow-ups on larger samples are needed to confirm the efficacy of PRP for the treatment of KOA. When employed in combination therapy, the joint use of PRP and HA showed a marginal edge over the sole application of PRP, with an improved safety profile [151].

Some authors report potential benefits for pain and functional outcomes in KOA over long-term treatment with dextrose prolotherapy, which involves applying dextrose to OA sites to promote tissue regeneration [152,153], but existing studies are at high risk of bias [132,154]. Dextrose has been approved by the Food and Drug Administration for intravenous use [155], but not for prolotherapy. Thus, long-term studies with a low risk of bias are needed to confirm the efficacy and safety of dextrose prolotherapy in KOA.

An increasingly used non-pharmacological approach for pain control in KOA patients is genicular nerve radiofrequency ablation, which showed superiority to traditional treatments in randomized controlled trials [156]. However, larger studies are needed for more evidence and confidence and to explore additional sensory nerves for enhanced treatment success [156].

Table 2 summarizes the pros and cons of mini-invasive pharmacological and non-pharmacological treatments for KOA, along with opinions from some major scientific societies.

### 4.3. Combination Approaches in Knee Osteoarthritis Management

Combination therapies have been suggested to increase efficacy by targeting different pain mechanisms and to reduce adverse effects by reducing doses. However, the efficacy of a combination of different treatment approaches is only narrative or with low-grade evidence. NSAIDs can be combined either with central analgesics, such as paracetamol and opioids, reducing doses and adverse effects [64]. However, concerns have been raised regarding the use of paracetamol in KOA joint pain due to its aforementioned side effects, as well as potential interactions with eventual hypertensive therapy and uncertain effectiveness [119,157].

Combining NSAIDs with SYSADOAs is interesting for multimodal therapy. SYSADOAs serve as background therapy, and oral NSAIDs manage acute KOA symptoms and potentially slow disease progression [63].

For elevated pain levels, synovitis, or effusions, combining IA injections with oral NSAIDs is valuable for early rehabilitation [44]. The fast onset and residual analgesic effect of NSAIDs [158], along with the longer-lasting effects of IA products, such as HA [159] and glucocorticoids [160], are intriguing. SYSADOAs and HA combinations also hold potential.

### 4.4. Non-Conservative Management of Knee Osteoarthritis

#### 4.4.1. Surgical Approaches for Knee Osteoarthritis Management

Surgical treatment of KOA is generally considered only in case of symptom persistence after nonsurgical treatment and radiological evidence of advanced OA (KL grade ≥ 3) [161]. Despite the fact that patients with mild KOA (KL grade 2) are increasingly being referred for TKA due to the constant advancements in long-term outcomes and reduced employment of other surgical procedures such as osteotomy, dissatisfaction about the outcome is more common in these patients [162]. Consultation with an orthopedic and traumatology specialist is essential to determine surgical intervention’s suitability [64].

Surgical treatment options include arthroscopic debridement, cartilage repair surgery, osteotomy with axis correction, and unicompartmental (UKA) or total knee arthroplasty (TKA) [161]. The choice depends on factors, such as symptoms, OA location, stage, age, physical activity level, comorbidities, and patient suffering [161].

While arthroscopy has been widely used for KOA [163], there is a lack of evidence supporting its significant benefits [161]. It may provide short-term relief to selected patients with mild radiographic KOA and effusion but should not be used as a KOA treatment [161], with very few motivated exceptions.

Cartilage repair is only suitable for focal cartilage defects and is not recommended for extensive defects [161]. Techniques include bone marrow stimulating, replacement, and combined techniques [161].

Osteotomies are effective for treating unicompartmental KOA with associated varus or valgus deformity [161]. UKA or unloading osteotomy can be considered for patients with KOA limited to one compartment, particularly in young and active patients who tend to experience inferior outcomes with TKA [164,165]. However, some authors [166,167] suggest that UKA should be considered a definitive solution as much as possible and therefore offered to older patients with KOA limited to one compartment. In younger patients, UKA might be offered too early (overtreatment), exposing them to a predictable risk of future revision surgeries [168]. There is no consensus among surgeons on UKA, and although a 20% UKA/TKA rate has been indicated as the “correct” one [169], actual rates vary quite considerably across institutions [170].

Prosthetic components have limited durability, making them generally less preferable for patients younger than 60 [161]. TKA is highly effective in elderly patients with advanced KOA [161], significantly improving patient functioning and QoL [161,171] even in long-term follow-ups [172].

In some cases, postoperative pain can persist despite the absence of clinical or radiological abnormalities [173].

Table 4 summarizes the pros and cons of surgical approaches for the treatment of KOA, along with opinions from some major scientific societies.

#### 4.4.2. Challenges in Assessing the Appropriateness of TKA Utilization

The increasing use of TKA in the United States is fueled by growing demand and a larger pool of eligible patients [174]. Criteria for TKA appropriateness have been established by various countries [175,176,177,178], including the widely used algorithm developed by Escobar et al. [175].

A multi-center cohort study [179] reported approximately one-third of total TKAs deemed as inappropriate using Escobar’s criteria [175], primarily among patients with mild or moderate symptoms and KL scores ≤ 2 [175]. Results suggest that inappropriate selection for surgery leads to delayed substantial improvements with respect to patients deemed as appropriate or inconclusive [180]. Chronic post-operative pain following TKA affects approximately 10–34% of patients [173], challenging both patients and healthcare providers and being a major source of dissatisfaction [181]. While pain reduction is the primary TKA expectation, the increasing TKA utilization suggests that more patients will face chronic pain [182] which imposes economic costs and hampers work productivity [173]. The risk and intensity of post-operative chronic pain correlate with preoperative pain severity [173], chronic pain in other parts of the body that may influence the central modulation of pain [183,184] and comorbidities [185]. Notably, around 70% of TKA patients have at least one comorbidity [186].

A recent Italian study found dissatisfaction with the current Diagnosis-Related Groups system, which tends to favor prosthetic solutions over less invasive options for managing OA [187]. Surgeons noted higher reimbursements for hospitals performing joint arthroplasty, influencing treatment choices for OA patients [187]. The trend of expanding joint arthroplasty indications to younger patients and milder OA forms is linked to less favorable outcomes and increased revision surgery needs [187].

## 5. Expert Perspective on KOA Management in Italy

### 5.1. Expert Perspective on Personalized Conservative Management of Pain in KOA

Despite limited data, experts estimate that around 60% of Italians with KOA experience moderate to severe pain, especially those over 65 years old. GPs often underestimate KOA’s impact due to misconceptions about it being part of aging [66], affecting patient motivation and adherence to recommended first-line treatments, such as exercise therapy [188] and weight loss advice [189].

Experts recommend early diagnosis, which, in selected cases, can be supported by bone scintigraphy and the involvement of rheumatologists for inflammatory KOA and physiatrists for conservative interventions. Orthopedic surgeons should come into play later, especially for surgical candidates.

Experts underline that non-pharmacological interventions, such as nutritional and joint education, weight reduction, exercise, and physiotherapy, are crucial for pain relief and slowing disease progression. They highlight how a loss of ≥5 kg or a 10% weight loss reduces the risk of symptomatic KOA by 50% [190] and significantly alleviates pain [46,191,192,193], respectively. Greater weight loss enhances pain reduction [194]. Additionally, a 5% weight loss improves functional and clinical outcomes [58], and weight loss is crucial for favorable knee replacement surgery outcomes [195].

Improving access to early rehabilitative and non-pharmacological treatments is essential for maximizing effectiveness. This involves the early involvement of the physiatrist when osteo-cartilaginous damage is in its initial stages. Experts emphasize the importance of launching a disease awareness campaign not only targeted at patients but also at healthcare providers and the community, to educate them about the significance and impact of non-pharmacological interventions, including appropriate joint care, during the early stages of KOA. Understanding the etiology, risk factors (particularly those that can be modified), anticipated prognosis, and therapeutic strategies associated with OA can contribute to minimizing misunderstandings and errors in patients [62]. This solution is considered highly feasible, with experts recommending the creation of informational booklets or videos to serve as a helpful guide for patients.

The experts note that while available pharmacological therapies are useful for pain reduction, they often have limited duration, which does not align with patients’ high expectations. Paracetamol is effective for mild pain treatment. NSAIDs, beneficial for acute pain management, are restricted by side effects and organ toxicity in chronic treatments. Weak opioids (often combined with paracetamol) provide short-term efficacy with high interindividual variability, and strong opioids are effective for managing chronic pain but are less effective on mechanical pain with low tolerability.

As regards IA therapy, reliance on professional guidelines and clinical experience influence injection recommendations [196]. The experts deem corticosteroid-based IA therapy efficient in inflammatory OA, requiring a low number of IA injections and presenting a lower cost. However, it may have IA toxicity, contraindications, and an increased risk of infection, leading to the need for a three-month interruption of corticosteroids pre-surgery. In expert’s opinion, HA IA injections could be useful for long-term OA management, reducing the need for corticosteroids, but their efficacy varies with the disease stage and individual responsiveness. Drawbacks include the need for repeated injections, partial efficacy, slow onset, and a lack of reimbursement.

While it is established that IA with corticosteroids and HA have entirely different mechanisms of action, effects, and goals making them not interchangeable therapies, experts still report a lack of consensus in the literature regarding the number and optimal frequency of IA injections for both drugs. Typically, experts argue that cycles of 3 IA injections of HA are performed with a delay of 1–2 weeks between each cycle, for a maximum of 2 cycles per year. Optimal results are often achieved with individual IA injections, depending on the type of drug used, and it is necessary to consider the feasibility as well as the preferences of both the clinician and the patient regarding single injections. Concerns about corticosteroid injections described in the literature include systemic exposure in geriatric patients with multiple joint issues and a higher willingness to offer them to patients with advanced OA [196].

Experts view PRP injections as a possible alternative to HA, with a sometimes contradictory but increasing body of literature.

They highlight the potential of MSCs IA injection in osteoarthritis therapy, as reviewed by Zhu et al. in their study [147], though evidence in the literature is scarce, and costs are high.

Experts highlight that in Italy, IA injection therapies are usually prescribed and administered by orthopedists, in contrast to other countries, such as France, where rheumatologists are the primary specialists. However, they emphasize that the scarcity of rheumatologists in Italy, along with the lack of specialized training in OA for pain therapists, may limit IA injections. The inconsistency in access to various treatments, particularly IA therapies, significantly impacts the patient’s journey. Region and local disparities exist in the reimbursement policies, and the practicality of therapeutic solutions. Consequently, there is a risk of performing a treatment that might not be the most appropriate for the current stage of the disease. This situation may compel clinicians to guide patients toward private facilities to ensure they receive the most suitable treatment for their condition.

Experts highlight that IA therapies are not covered by the national health systems (NHS) and restructuring payment incentives to encourage conservative treatments is needed.

Additionally, studies show that injectables not covered by insurance or on the formulary are less likely to be used [196,197]. However, in some cases, injections are preferred over higher-cost treatments, such as physical therapy, highlighting the role of the reimbursement system in influencing treatment choices [187,196]. In other cases, however, injection may be preferred over physical therapy due to minimal time involvement and no required lifestyle changes. Thus, experts highlight that it is crucial to take time and explain the uncertain benefits of injections and the limits of their results without a concomitant rehabilitative approach.

In response to the ambiguity and inconsistency in access to IA therapies, experts suggest the establishment of a national interdisciplinary consensus. This consensus would aim to elucidate the specialists responsible for prescribing and administering IA therapies and determine the most suitable stage in a patient’s KOA treatment journey for their use, thus ensuring patients are directed effectively.

Implementing these solutions can serve as an initial stride towards establishing a comprehensive, multidisciplinary approach program providing continuous guidance and support to patients throughout their entire journey.

Experts suggest that a new IA treatment for KOA patients should have an effect exceeding 20% in comparison to existing therapies, making it potentially reimbursable by the NHS. A single administration would improve both therapy’s logistics and patient acceptance and should not interfere with surgical timing.

A recent expert opinion paper outlined ambitious targets for an innovative IA HA product to surpass current clinical benefit standards for mild-to-moderate KOA, such as aiming for a 50–70% improvement in nociceptive pain and function in at least 70% of patients and maintaining benefit for 9–12 months [198]. The paper suggests that early benefits could be valuable, particularly for patients with comorbid conditions [198]. Crucially, the product should cause local reactions in fewer than 5% of patients and exhibit no systemic adverse effects, even with multiple injections [198].

### 5.2. Expert Perspective on Dissatisfaction among Healthcare Providers and KOA Patients

The experts highlight the existing discrepancy between the expected outcomes of pain reduction therapy. Indeed, they consider a 50% reduction in pain as an excellent outcome, but patients have higher expectations. Dissatisfaction with pain relief achieved with current treatment plans has been reported among 26.8% to over 50% of patients across different studies [199,200], highlighting the demand for new OA medications [200].

Adverse effects concern OA patients, with risk tolerance varying based on symptom type and baseline levels [201] and older individuals prioritizing fewer adverse events over higher treatment efficacy [202]. Patients may prefer exercise over prescription treatments due to aversion to adverse event risks [203]. Gastrointestinal issues are prevalent, likely due to high NSAID use [204].

Patient expectations impact GPs’ treatment choices [66], sometimes leading to unnecessary tests for trust maintenance [205]. In other cases, patients’ expectations may not be considered at all [78,79]. Understanding patient-preferred outcomes, such as reducing ambulatory pain and difficulty in daily activities [201], is crucial for satisfaction and participation.

Nearly half of Global Osteoarthritis Patient Perception Survey respondents reported reduced QoL [200], considering lower expectations in elderly patients [206]. Almost 95% expected improved satisfaction if OA were eliminated, highlighting the urgency for better management strategies [200].

Experts stress the importance of clear communication from healthcare providers regarding expected surgical outcomes and the possibility of enduring chronic pain. The experts participating in this study estimate that around 25% of patients, mainly patients aged 55–65 years with KL grade 3, may experience chronic pain and dissatisfaction following surgery even when no clear problem can be ascertained. These patients, often with limited therapeutic options due to their age, inadequate response to pharmacological therapy, and high functional requirements, may have high expectations for surgery and a higher likelihood of dissatisfaction with outcomes. Conversely, individuals with severe comorbidities or diminished physical function may be considered ineligible for TKA, even if it is necessary, primarily due to anesthesia concerns. Cultural considerations may lead to surgery refusal in some cases. Lack of clear communication is a common issue, with over half of the patients in the Global Osteoarthritis Patient Perception Survey expressing dissatisfaction with their doctors’ explanations about their OA diagnosis and treatment options [200].

Waiting times for surgery in Italy range from 1 to 24 months, including affiliated private centers, with larger centers estimating a minimum of 4 months for knee replacement. It is crucial for orthopedic practitioners to employ strategies for pain management during the waiting period to prevent the escalation of chronic pain, potential sedentary behavior, muscle hypotonia, and persistent discomfort post-surgery, which may hinder the recovery process after TKA, particularly in older patients.

## 6. Strategies for Improving Knee Osteoarthritis Patient Management

Promoting educational programs for healthcare professionals and patients can enhance awareness and proactive management [66], reducing diagnostic delays [65]. GPs should receive training in effective osteoarthritis management, including active patient engagement and staying updated on osteoarthritis research [65]. Enhancing GP training in early KOA, phenotype recognition using biomarkers, and imaging technologies such as MRI is vital for accurate referrals to specialists. Experts stress the need for personalized treatment in moderate to severe KOA pain, considering factors, such as age, comorbidities, and pain severity.

## 7. Future Perspectives

### 7.1. Treat-to-Target Treatment of KOA

KOA’s heterogeneous presentation by phenotype and disease stage complicates structural target identification for early multimodal interventions [115]. Emerging research priorities to devise appropriate treat-to-target strategies, according to Migliore et al. [115], include discovering specific disease progression biomarkers, identifying suitable physical exercise for different KOA phenotypes, defining optimal BMI ranges, exploring comorbidity interactions, characterizing individual pain characteristics, and utilizing digital tools for long-term KOA management.

### 7.2. Innovative Therapies for KOA Management

Emerging strategies for IA steroid treatments include sustained-release corticosteroid formulations [207,208,209,210], and hydrogel-based steroid combination therapies [211,212], aiming for lasting effects and reduced need for frequent injections [211]. Innovative HA preparations with optimal viscosity or antioxidants aim to slow HA degradation and reduce inflammation for sustained pain relief [130].

Clodronate IA injections, reducing joint inflammation, may be a beneficial approach to KOA potentially synergizing with HA injections, even if more studies exploring this combined approach are needed [130]. Combination therapy with collagen and HA is being explored, given their comparable efficacy [130].

Autologous chondrocyte implantation (ACI) and matrix-assisted chondrocyte implantation (MACI), effective for treating focal chondral lesions [213,214,215], are now suggested for regenerating damaged cartilage in early KOA [216] and delaying joint replacement in younger patients [217]. Despite concerns about the inflammatory environment hindering regeneration [218,219], autologous chondrocytes from OA patients have shown superior behavior, surpassing donor-matched MSCs [220].

While achieving long-term stable results is challenging due to the progressive degenerative nature of KOA [221], recent reviews indicate promising mid- and long-term improvements with both techniques [213,216].

Adipose, bone marrow, or umbilical cord-derived MSCs exhibit therapeutic potential for pain reduction and joint improvement [147], but larger clinical trials with longer follow-ups are necessary for confirmation.

Structure-modifying drugs, such as sprifermin, a recombinant human FGF18 [222,223], and lorecivint [224,225] displayed potential effectiveness in improving joint structure and reducing cartilage loss but did not provide clear clinical benefits specifically in pain reduction [226,227]. Other molecules interacting specifically with chondrocytes, such as LNA043 [228,229,230] and TPX-100 [231,232], showed cartilage regeneration potential and improvement of pain and function, respectively.

Gene therapy, involving therapeutic genes delivered to joint cells, has also shown promise [233], with safety, tolerability, and pain relief [234,235].

Nonetheless, pain is the most frequent symptom reported by KOA patients and has a significant impact on their QoL. Therefore, great research efforts are being directed towards the improvement of the control of pain, as it is the primary and most invalidating symptom of KOA. The transient receptor potential vanilloid-1 (TRPV-1) channel is a compelling target currently under active clinical investigation for treating KOA pain. Predominantly expressed by sensory neurons [236], TRPV-1 is a ligand-gated, non-selective cation channel responsive to various noxious stimuli, such as intense heat, acid, and exogenous agonists [237]. Pharmacological strategies for pain management have explored both TRPV-1 agonists and antagonists [236]. Exogenous agonists can induce long-term effects on sensory nerve fibers through mechanisms such as desensitization, nociceptor dysfunction, neuropeptide depletion, and reversible nerve fiber defunctionalization [238]. Antagonists, on the other hand, directly block the function of TRPV-1 itself [237], but currently, no TRPV1 antagonists are approved for human use. All those who have undergone clinical trials experienced significant systemic adverse events, including impairment of heat perception, leading to their discontinuation [239]. TRPV-1 agonists have undergone clinical trials, demonstrating promising effects on pain reduction [236]. Preliminary results report mild adverse events, such as a burning sensation with topical TRPV-1 agonists or pain, tachycardia, and hypertension with IA TRPV-1 agonists [236].

## 8. Conclusions

The complex nature of KOA poses challenges in developing pharmaceutical treatments that effectively address structural changes and especially pain relief. IA therapies seem suitable for KOA, but better drug targets and a more targeted approach based on disease stage, causes, and risk factors are needed. Tailoring treatments to individual patients’ conditions and goals is essential. Discussions and comparisons among experts have revealed a significant lack of consistency in the care and treatment of KOA patients in Italy, which makes the patients’ journey erratic and inconclusive. This heterogeneity can be attributed to variations in clinical experiences and approaches among healthcare providers, concerns over side effects and affordability in an aging population with KOA, disparities in regional and local healthcare systems, and the absence of a well-defined and universally accepted pathway that specifies when and how different specialists should intervene in managing KOA patients. Consequently, the optimal solution, albeit a challenging one to implement, would involve establishing a multidisciplinary approach program that encourages interaction and collaboration among various specialists.

## Figures and Tables

**Table 1:** Topical and oral pharmacological treatments in KOA patients without comorbidities: pros, cons, and guideline recommendations. Source: Original.

Topical and Oral Pharmacological Treatments	Level of Recommendation *
Paracetamol	
Pros Provides central analgesia for mild pain Cons Less effective as an analgesic compared to opioids and has a lower anti-inflammatory effect than NSAIDs Associated with safety concerns	OARSI [56]: No recommendation ESCEO [55]: No recommendation ACR/AF [58]: Conditional EULAR [60]: No recommendation NICE [61]: NOT recommended SIOT [62]: No recommendation
Oral SYSADOAs (GlcN and CS)	
Pros Long-lasting effects after therapy Slows the progression of OA Reduces the required dosage of NSAIDs Cons No immediate analgesic properties Effects are delayed, taking 2–3 weeks to manifest	OARSI [56]: Conditional ESCEO [55]: Strong (pharmaceutical grade) ACR/AF [58]: Strong against EULAR [60]: A NICE [61]: No recommendation SIOT [62]: Weak
Topical NSAIDs	
Pros Suitable for individuals with symptomatic KOA Minimal to mild side effects Cons High cost	OARSI [56]: Strong ESCEO [55]: Recommended (if symptoms persist after short-term paracetamol SYSADOAs, and exercise) ACR/AF [58]: Strong EULAR [60]: A NICE [61]: Recommended SIOT [62]: Strong
Oral NSAIDs	
Pros Effective for persistent symptoms when SYSADOAs are ineffective Cons Risk of toxicity with long-term use	OARSI [56]: Conditional ESCEO [55]: Strong ACR/AF [58]: Strong EULAR [60]: A NICE [61]: Recommended after topical treatment failure SIOT [62]: Recommended
Weak opioids	
Pros Effective in the short-term Cons High variability in response between individuals Can cause constipation Concerns about tolerability and dependance	OARSI [56]: Conditional ESCEO [55]: Recommended short-term use ACR/AF [58]: Conditional against non-tramadol opioids and conditional for tramadol EULAR [60]: B NICE [61]: Recommended only for short term relief after failure of all other pharmacological options SIOT [62]: Recommended for short term therapy before surgery
Duloxetine	
Pros Suitable for widespread pain and central sensitization as an alternative to weak opioids	OARSI [56]: Conditional ESCEO [55]: Weak ACR/AF [58]: Conditional EULAR [60] B NICE [61]: No recommendation SIOT [62]: Conditional

NSAIDS; non-steroidal anti-inflammatory drugs; KOA: knee osteoarthritis; GlcN: glucosamine; CS: chondroitin sulfate; pGCS: patented crystalline GlcN; SYSADOAs: Symptomatic Slow-Acting Drugs for Osteoarthritis; OARSI: Osteoarthritis Research Society International; ESCEO: European Society for Clinical and Economic Aspects of Osteoporosis, Osteoarthritis, and Musculoskeletal Diseases; ACR: American College of Rheumatology; AF: Arthritis Foundation; EULAR: European League Against Rheumatism; NICE: National Institute for Health and Care Excellence. * In the absence of comorbidities.

**Table 2:** Minimally invasive pharmacological and non-pharmacological therapy in KOA patients without comorbidities: pros, cons, and guideline recommendations. Source: Original.

Minimally Invasive Pharmacological Therapy	Level of Recommendation *
IA treatments	
Pros Enhanced local bioavailability Minimized systemic exposure Optimal results with individual IA injections Reduced frequency of adverse effects No systemic or severe adverse events Cons Limited effectiveness in severe forms of OA Requires specific skills and experience; shortage of skilled specialists Lack of consensus on the optimal number and frequency of IA injections Necessitates strict aseptic procedures Inconsistent access to treatment	OARSI [56]: Conditional ESCEO [55]: Weak ACR/AF [58]: See recommendation for specific IA drugs EULAR [60]: See recommendation for specific IA drugs NICE [61]: See recommendation for specific IA drugs SIOT [62]: See recommendation for specific IA drugs
Anti-inflammatory and analgesic IA therapies:	
IA corticosteroids	
Pros Potent anti-inflammatory properties Rapid pain relief Efficacy in cases of synovitis Requires a low number of IA injections Lower cost Cons Short-term therapeutic effect Risks associated with long-term use Systemic exposure risk in geriatric patients with multiple joint issues Needs to be discontinued before surgery	OARSI [56]: Conditional ESCEO [55]: No recommendation ACR/AF [58]: Strong EULAR [60]: A NICE [61]: Recommended for short-term relief after failure of other treatments failed or to support therapeutic exercise SIOT [62]: Recommended for short-term use
IA HA	
Pros Favorable long-term safety profile Cons Efficacy varies depending on the disease stage Requires repeated injections Slow onset of action Lack of reimbursement	OARSI [56]: Conditional ESCEO [67]: Recommended ACR/AF [58]: Conditional against EULAR [60]: B NICE [61]: Not recommended SIOT [62]: Recommended
IA Ozone and botulinum toxin	
Pros Anti-inflammatory and analgesic properties High safety profile Cons Insufficient evidence to support effectiveness	OARSI [56]: No recommendation ESCEO [55]: No recommendation ACR/AF [58]: Conditional against for botulinum toxin EULAR [60]: No recommendation NICE [61]: No recommendation SIOT [62]: Limited recommendation for ozone therapy
Regenerative IA therapies:	
IA MSCs	
Pros Potential for pain reduction and joint improvement Cons Limited evidence of effectiveness High costs Requires further clinical trials	OARSI [56]: No recommendation ESCEO [67]: No recommendation ACR/AF [58]: Weak EULAR [60]: No recommendation NICE [61]: No recommendation SIOT [62]: Strong against
IA PRP	
Pros Valid treatment for pain reduction and functional improvement Cons Limited evidence available Requires longer follow-up studies	OARSI [56]: No recommendation ESCEO [67]: No recommendation ACR/AF [58]: Strong against EULAR [60]: No recommendation NICE [61]: No recommendation SIOT [62]: Conditional
Dextrose prolotherapy	
Pros Pain reduction Functional improvement Cons More evidence is needed	OARSI [56]: No recommendation ESCEO [55]: No recommendation ACR/AF [58]: Conditional against EULAR [60]: No recommendation NICE [61]: No recommendation SIOT [62]: No recommendation
**Minimally Invasive Non-Pharmacological Therapy**	Level of Recommendation *
Genicular Nerve Radiofrequency Ablation	
Pros Superior to IA corticosteroids, HA, and oral analgesics. Favorable safety profile	OARSI [56]: No recommendation ESCEO [55]: No recommendation ACR/AF [58]: Conditional EULAR [60]: No recommendation NICE [61]: NOT recommended SIOT [62]: No recommendation

IA: intra-articular; PRP: platelet-rich plasma; MSCs: mesenchymal stem cells; OARSI: Osteoarthritis Research Society International; ESCEO: European Society for Clinical and Economic Aspects of Osteoporosis, Osteoarthritis and Musculoskeletal Diseases; ACR: American College of Rheumatology; AF: Arthritis Foundation; EULAR: European League Against Rheumatism; NICE: National Institute for Health and Care Excellence. * In the absence of comorbidities.

**Table 4:** Surgical therapy in KOA patients without comorbidities: pros, cons, and guideline recommendations. Source: original.

Surgical Therapy	Level of Recommendation *
Arthroscopy	
Pros Potential for short-term relief in mild cases Cons Inadequate evidence for substantial benefits Not suitable as a routine treatment	OARSI [56]: No recommendation ESCEO [55]: No recommendation ACR/AF [58]: No recommendation EULAR [60]: C NICE [61]: NOT recommended SIOT [62]: No recommendation
Cartilage repair	
Pros Suitable for focal cartilage defects Cons Not recommended for extensive cartilage defects	OARSI [56]: No recommendation ESCEO [55]: No recommendation ACR/AF [58]: No recommendation EULAR [60]: No recommendation NICE [61]: No recommendation SIOT [62]: No recommendation
Osteotomies	
Pros Effective for treating unicompartmental KOA with deformity Cons Limited to young and active patients	OARSI [56]: No recommendation ESCEO [67]: No recommendation ACR/AF [58]: No recommendation EULAR [60]: C NICE [61]: No recommendation SIOT [62]: No recommendation
TKA	
Pros Commonly used for elderly patients with advanced KOA Significant improvement in patient functioning and QoL Cons Possibility of persistent chronic pain Risk of implant failure, which may necessitate revision surgery Inferior outcomes in younger patients.	OARSI [56]: No recommendation ESCEO [67]: Strong for end-stage KOA ACR/AF [58]: No recommendation EULAR [60]: C NICE [61]: Recommended only in case of substantial impact on QoL and when non-surgical treatments failed SIOT [62]: No recommendation

TKA: total knee arthroscopy; KOA: knee osteoarthritis; ESCEO: European Society for Clinical and Economic Aspects of Osteoporosis, Osteoarthritis, and Musculoskeletal Diseases; EULAR: European League Against Rheumatism; NICE: National Institute for Health and Care Excellence. * In the absence of comorbidities.

## Data Availability

The raw data supporting the conclusions of this article will be made available by the authors upon request.
